# Endo-Bentall Repair for Inoperable Aortic Disease: Balancing Innovation and Ethical Responsibility

**DOI:** 10.1016/j.ejvsvf.2026.06.004

**Published:** 2026-06-23

**Authors:** Geoffrey Ying, Niklas Juth, Anders Wanhainen, Rickard Lindblom, Stefan James, Carlota Fernandez Prendes, Kevin Mani

**Affiliations:** a Department of Surgical Sciences, Vascular Surgery, Uppsala University, Uppsala, Sweden; b Department of Surgery, Auckland City Hospital, Auckland, New Zealand; c Department of Public Health and Caring Sciences, Centre for Research Ethics and Bioethics, Uppsala University, Uppsala, Sweden; d Department of Diagnostics and Intervention, Umeå University, Umeå, Sweden; e Department of Surgical Sciences, Cardiothoracic Surgery, Uppsala University, Uppsala, Sweden; f Department of Medical Sciences, Cardiology, Uppsala University, Uppsala, Sweden

**Keywords:** Aortic root replacement, Ascending aortic repair, Compassionate use, Endovascular aortic repair, Innovative cardiovascular therapy, Medical ethics

## Abstract

**Objective:**

To evaluate the ethics of introducing the Endo-Bentall (a novel high risk procedure) in the context of compassionate care and to make practical recommendations to support its incorporation into practice.

**Methods:**

A seven step ethical framework was systematically applied to three clinical vignettes involving the Endo-Bentall procedure. Ethical issues identified across cases were synthesised and used to develop practical recommendations for clinical practice.

**Results:**

Decision making regarding the Endo-Bentall procedure is challenging in the absence of robust outcome data. Patients amenable to treatment represent a highly heterogeneous population, with a high degree of variability in procedural risk, likelihood of technical success, consequences of failure, and uncertainty of long term durability. Despite this, ethical justification may be achievable in carefully selected cases where anticipated benefits proportionately outweigh risks, particularly in the context of otherwise limited therapeutic alternatives.

**Conclusion:**

Ethical implementation of the Endo-Bentall procedure is possible in select patients. However, structured safeguards are necessary. These include enhanced informed consent processes with explicit disclosure of the learning curve, limited validation, and unknown long term outcomes; mitigation of potential conflicts of interest through independent or external case review; formal oversight mechanisms despite its classification as compassionate or innovative care; and integration within a learning health system framework with ongoing data reporting and outcome monitoring.

## Highlights

• Unrestricted surgical innovation can result in significant harm.
• Not all patients are ethically suitable as compassionate cases for novel procedures.
• Balancing risk and benefit is challenging in the absence of robust outcome data.
• Third party input is essential for decisions on novel high risk procedures.
• Addressing threats to ethical practice requires mandatory oversight.

## Introduction

**Figure 1:**
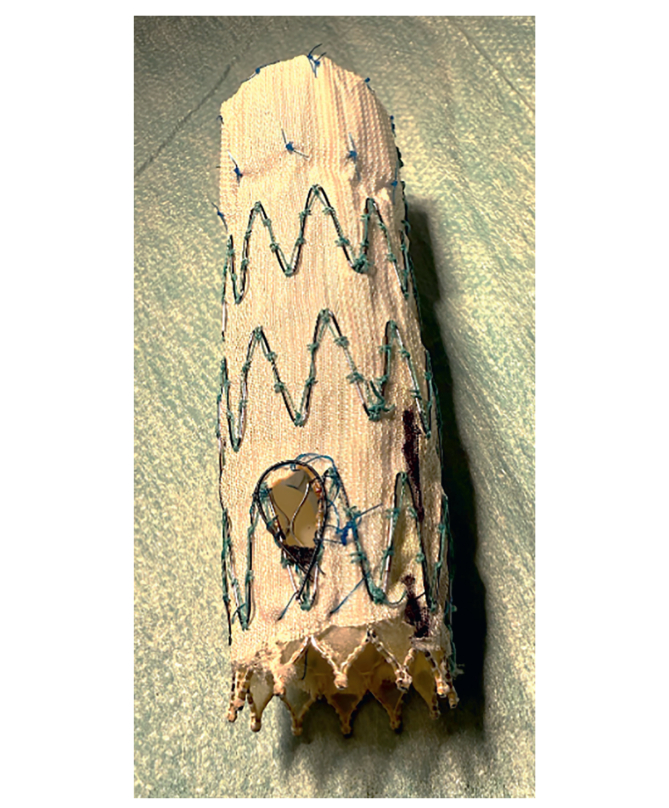
Example of an Endo-Bentall prosthesis consisting of a physician modified thoracic endograft combined with a transaortic valve replacement endoprosthesis, including pre-made fenestrations for the coronary artery ostia.

**Figure 2:**
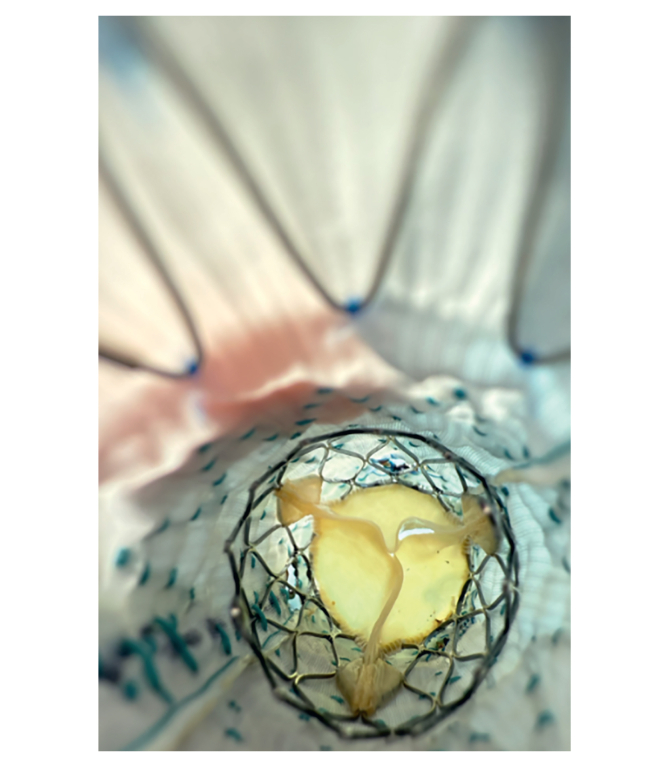
Internal view of a combined aortic valve and ascending stentgraft Endo-Bentall prosthesis.

The evolution of aortic endovascular therapies has enabled the treatment of increasingly complex aortic pathology and anatomy. The aortic root remains one of the few areas still requiring open surgery. However, this too may be changing. The Endo-Bentall uses a physician constructed device consisting of a self expanding transcatheter aortic valve sutured into a thoracic endovascular stent graft, enabling the exclusion of both the aortic root and ascending aorta. Coronary stents are deployed through either fenestrations or branches within the endograft to maintain coronary perfusion (Figure 1, Figure 2). When a seal at aortic valve level is not required, such as in aortic dissection with an entry tear distal to the sinotubular junction, the EndoWheat procedure may be used instead.^1^ This approach similarly combines transcatheter aortic valve replacement (TAVR) and endovascular thoracic stent grafting, although coronary revascularisation is not required as the native aortic root and coronary ostia are preserved either through unstented fenestrations or an uncovered gap between the TAVR and ascending endograft (Figure 1, Figure 2).

The natural history of aortic root and ascending arch pathologies such as aneurysm or dissection can be catastrophic if untreated. The Endo-Bentall and EndoWheat would therefore be invaluable treatment options for patients with prohibitive risk of open surgery. However, data are scant, and little is known about the efficacy and safety of these procedures. This is an area of significant concern in the realm of endovascular surgery where high risk devices such as the Nellix system (Endologix Inc., Irvine, CA, USA) have previously been allowed to enter the market prematurely, resulting in unacceptably high failure rates.^2^, ^3^, ^4^, ^5^ Risk is further amplified in interventions to the aortic root and ascending aorta. Proximity to the heart creates dynamic multi-axial deformations in the ascending aorta with length and diameter changes, phase shifting between cyclic deformations and alternating twist during each cardiac cycle.^6^ These biomechnical forces become more pronounced as the aortic root is approached, creating a hostile environment for endovascular intervention.^7^ More proximal intervention carries a progressively greater risk of stroke and a higher 30 day mortality rate,^8^^,^^9^ now potentially with the added complexity of coronary revascularisation where graft malpositioning or migration could be catastrophic. Caution is therefore warranted to ensure that procedural innovation is guided by robust evidence and appropriate ethical scrutiny. Typically, these novel, unproven procedures are reserved for compassionate use.^10^^,^^11^ However, this invariably begs the question of how compassionate use is defined, the ethical principles that should guide such decisions, and the potential conflicts that may arise.

The aim of this article is to address the challenges of implementing combined endovascular aortic root and ascending aortic repair in an advanced societal healthcare system and to determine how this might be done, if at all, from an ethical perspective. For the purpose of this paper, the term Endo-Bentall is used, but the same ethical considerations apply to the EndoWheat procedure. These considerations are demonstrated and evaluated using three clinical scenarios.

## Materials and methods

The analysis uses a seven step process used by Miljeteig *et al,*^12^^,^^13^ which is summarised in Table 1.

**Table 1:** Ethical case analysis of the introduction of a new technique in healthcare using a seven step approach.

1.	Statement of the problem and alternative actions or rules
2.	What is the evidence concerning the outcomes of the different alternatives?
3.	Are there guidelines or legal acts that regulate the issue at hand?
4.	Who are the affected parties?
5.	What are the benefits and burdens for the affected parties?
6.	Are substantial interests in conflict?
7.	Are fundamental principles in conflict?

As regards the substantial ethical principles involved in the analysis, the four principles of medical ethics are used: beneficence, non-maleficence, autonomy, and justice.^14^ The analysis will be applied to the three following clinical scenarios. These will be used to guide the discussion.

### Patient A

A 68 year old woman is known to have an asymptomatic ascending aortic aneurysm of 6.5 cm, enlarging by 1 – 2 mm a year during follow up. She has a background of chronic kidney disease and chronic obstructive pulmonary disease and is unsuitable for open repair owing to the high risk of respiratory and chronic kidney disease related complications. Endo-Bentall is thought to be technically feasible using the left ventricular outflow tract as a proximal landing zone for a total endovascular exclusion of the aneurysm.

### Patient B

An 81 year old man presents with acute type A aortic dissection. He has a history of myocardial infarction with previous coronary artery bypass grafting. He currently has no angina, and echocardiogram demonstrates a left ventricular ejection fraction of 50%. He also has grade II chronic obstructive pulmonary disease. This patient is regarded as unsuitable for open repair owing to age, comorbidities, and high risk of redo sternotomy and is thus managed medically despite the high risk of dissection related complications and death. He is alive twelve hours after dissection and has developed a minor pericardial effusion with no haemodynamic effect. The entry tear 1 cm above the sinotubular junction is thought to be amenable to treatment with the Endo-Bentall procedure.

### Patient C

A 78 year old woman with a previous ascending aortic repair complicated by a pseudoaneurysm at the proximal anastomosis. Follow up has shown enlargement from 34–75 mm over a two year period, although she is asymptomatic. The pseudoaneurysm is right against the sternum with a high risk of major haemorrhage if redo sternotomy is attempted, hence not suitable for open repair. Endovascular repair is possible but would require landing the stent graft in the valve.

## Analysis

### Statement of the problem and alternative actions/rules

Should the Endo-Bentall procedure be used in scenarios such as those described above?

### What is the evidence concerning the outcomes of the different alternatives?

Aortic root pathology amenable to treatment with the Endo-Bentall encompasses a spectrum of clinical conditions with variable outcomes. For aneurysms, the treatment threshold is in the range of 5.0 – 5.5 cm as per the American College of Cardiology (ACC) and American Heart Association (AHA) guidelines, and enlarging size is associated with an exponential increase in the risk of complications.^15^ Conversely, acute type A aortic dissection is a surgical emergency, with medical management alone carrying a two to threefold higher mortality rate than surgical management, and an hourly mortality rate of 1%.^15^ Emergency repair of rupture or dissection carries a much higher risk of death and morbidity than elective repair.^16^^,^^17^ Other pathologies include traumatic injury, anastomotic leak after surgery, and mycotic aneurysm, although these must be assessed on a case by case basis.

There are no universally accepted objective criteria that define fitness for open aortic root or arch surgery. Risk assessment entails a multidisciplinary team (MDT) discussion involving cardiothoracic surgeons, anaesthetists, intensivists, and other relevant specialists.^15^ Risk prediction tools such as the Arch Reconstruction under Circulatory arrest with Hypothermia score also exist, although there is no specific threshold that constitutes prohibitive risk.^18^ Consequently, management ultimately depends on clinical judgement informed by patient factors, anatomic considerations, and multidisciplinary consensus.

Data pertaining to endovascular treatment of the aortic root are limited. Currently, there exist only a limited number of case reports of the Endo-Bentall procedure being successfully used in humans.^10^^,^^19^, ^20^, ^21^ These isolated case reports have a high risk of selection and publication bias and lack data on long term outcomes. Assessment using the Grading of Recommendations, Assessment, Development and Evaluation system, therefore, suggests very low certainty of evidence, and evaluation using the Methodological Index for Non-Randomised Studies Quality Assessment Tool indicates poor methodological quality.^22^ Other data are limited to proof of concept studies and assessments of anatomic suitability. It is therefore impossible to comment accurately on the expected outcomes of patients treated with the Endo-Bentall.

### Are there guidelines or legal acts that regulate the issue at hand?

There are no established guidelines to aid decision making.

Legally, the following considerations are widely relevant to contemporary healthcare models in developed societies:^23^, ^24^, ^25^, ^26^, ^27^ (1) clinical research is subject to more stringent review than compassionate care often requiring approval from ethical boards; (2) the caregiver must respect the patient’s integrity, autonomy, and right to participation in decision making. No care must be performed without the patient’s consent; (3) practitioners are obliged to practice within their scope to deliver evidence based and patient centred care; (4) prioritisation should be based on the principle of human dignity, the principle of need and solidarity, and finally, the principle of cost effectiveness (these principles are then a specification of the general principle of justice); and (5) handling of patient data is carefully regulated.

### Who are the affected parties?

The stakeholders when considering the Endo-Bentall procedure include the patient, the patient’s close ones, the surgeon or clinician performing the procedure, the MDT involved, other patients requiring the same resources, future patients who may benefit, the hospital, and medical device companies.

### What are the benefits and burdens for the affected parties?

To the patient, the benefit is unclear in the absence of information to guide decision making. Intervention may result in the prolonging of life or increased survival. However, this is speculative and must be balanced against an unknown degree of peri-operative morbidity, death and late complications.

The patient’s close ones typically have potential benefits and burdens analogous to the patient and may need to serve as the patient’s representative in case the patient is no longer decision competent.

The surgeon stands to benefit if the Endo-Bentall is successful. Treatment of a patient is rewarding in and of itself. Furthermore, to pioneer a new technique suitable for a group of patients previously unfit for intervention would bring substantial professional recognition. The institution benefits similarly in the form of increased funding and enhanced reputation. However, both must bear in mind the potential professional scrutiny, and medicolegal repercussions for failing to adhere to ethical principles.

Other health professionals are also implicated. The MDT are responsible for deciding what constitutes prohibitive risk which can be challenging and subject to undue influence. Other specialists (anaesthetist, intensivist, nursing, etc.) must support the endeavour both ethically and practically.

The resources required to attempt the Endo-Bentall may result in delays and cancellations for other patients awaiting proven intervention. Conversely, future patients may benefit greatly. Medical device companies stand to profit if the procedure proves to be successful.

### Are substantial interests in conflict?

Conflict can occur at multiple levels. Decision making should be a shared process between patient and clinician. However, differing perspectives or understanding of acceptable risk and benefit can lead to discord regarding whether to proceed. Tensions can develop between the surgeon and the MDT who may withhold support owing to practical or ethical concerns. The hospital system may deem the risk excessive, potentially leading to feelings of undue restriction of potentially lifesaving treatment. These conflicts are further complicated by legal and reputational concerns, and potential biases driven by the desire to pioneer novel techniques. Resource allocation is an important consideration, because directing limited healthcare resources towards a high risk, unproven intervention may disadvantage other patients. Moreover, financial incentives tied to medical device manufacturers can create pressures beyond evidence based practice that unjustly influence clinical decisions.

### Are fundamental principles in conflict?

#### Autonomy

In healthcare, the principle of autonomy is often practiced through informed consent. Patients should understand the risks, benefits, and alternatives to any procedure being offered and make decisions based on this information. This is challenging as little is known about the efficacy or durability of the Endo-Bentall. Furthermore, patients faced with acute, life threatening illnesses constitute a vulnerable population where decisions may be guided by hope or desperation.^28^

#### Beneficence and non-maleficence

It is difficult to comment on the principles of beneficence and non-maleficence in the absence of robust data. Broadly speaking, the likelihood of success with good long term outcomes is unknown and the potential for harm is likely to be high, especially in asymptomatic patients. However, there is significant heterogeneity in patients suitable for treatment with the Endo-Bentall, as exemplified by the different clinical scenarios provided. Therefore, it is likely that a point exists along the spectrum where the combination of disease severity and predicted likelihood of success allows the reconciliation of beneficence and non-maleficence.

#### Justice

Allocation of resources towards high risk interventions with uncertain benefit can be seen as unjust because it deprives or delays treatment of other patients who likely have better prognoses. Although need trumps cost effectiveness when prioritising patients in most systems, the current level of evidence makes justifying use of the Endo-Bentall difficult. However, the development of a new method that later proves successful may well justify an initial resource re-allocation.

Another consideration is the patient selection process. In the absence of established guidelines, this responsibility falls solely on the treating clinician, raising potential ethical concerns. Prohibitive risk for open surgery is inherently subjective and susceptible to bias. Furthermore, the lack of standardised criteria for anatomic suitability and other predictors of success limit the reproducibility and fairness of patient selection.

## Discussion

Patient A has an ascending aortic aneurysm at the treatment threshold. Although Endo-Bentall is technically feasible, she remains asymptomatic. Therefore, a successful procedure would produce no benefit in quality of life and may not even alter her prognosis given the absence of long term data. Although the yearly risk of rupture or dissection is not insignificant, this is not absolute. A prophylactic procedure such as the Endo-Bentall is only beneficent if the risk of the procedure is lower than that of the natural disease history which cannot be deduced. Conversely, a failed or complicated procedure could significantly impair quality of life or end it prematurely. Therefore, it can be stipulated that the risk benefit balance is so unsure and potentially negative that the offer should not be made at all.

Patient B lies on the other end of the spectrum with an acute presentation and a high likelihood of death if untreated. Successful treatment would result in an immediate, tangible benefit in the form of prolonging life, even if longer term durability is poor. Similarly, the risk of causing more harm than conservative management is low given the poor prognosis. However, consideration must be extended to other factors. It is known that acute surgery is much riskier than elective surgery, with greater peri-operative morbidity and risk of death. A heroic attempt at salvage may be a futile, resource wasting endeavour. Furthermore, emergency situations have the potential to pressure patients, threatening autonomous decision making. Palliative care can provide comfort and dignity at the end of life, and patients must be allowed to recognise it as an appropriate option. However, in contrast to patient A, where the potential harm outweighs any ethical justification for the Endo-Bentall, patient B presents a different scenario: intervention can be justified provided safeguards are in place.

Patient C has a rapidly expanding pseudoaneurysm carrying a significantly higher risk of imminent rupture than that of a true aneurysm. Therefore, successful treatment would generate comparatively greater benefit than in patient A despite both being asymptomatic. The procedure can be carried out electively, increasing the likelihood of success and enhancing informed decision making. Moreover, because the patient is excluded from open surgery owing to technical factors rather than comorbidities, they are more likely to have a successful recovery. This scenario appears to appropriately balance ethical principles, maximising the probability of success while maintaining informed decision making.

The purpose of these cases is not to say definitively whether each individual should be offered treatment, rather to suggest that presentations lie on a continuum. From this analysis, it can be elucidated that there is an ethically justifiable role for the use of Endo-Bentall in select patients. Some practical recommendations in relation to the ethical aspects of such a novel procedure to address the challenges raised in this article are provided below.

### Informed consent

Proper informed consent is essential. Information provided must entail the innovative nature of the procedure, the risks and benefits of the procedure, potential unforeseeable complications, and alternatives.^29^, ^30^, ^31^, ^32^ Discussion must be balanced and not overestimate the likelihood of success. In addition to providing adequate and accurate information, other challenges include ensuring adequate patient comprehension and overcoming power dynamics, language barriers, and culture barriers.^33^ Suggested methods to address this include interactive consent, involvement of an impartial third party or /advocate, and allowing the patient a cooling off period to reflect on their decision.^30^

### Conflicts of interest

Safeguards need to be put in place to protect patients from potential conflicts of interest. Especially when the treating clinician stands to benefit from performing the procedure, decision making should be done in conjunction with independent experts to ensure that patients are selected based on clinical merit alone.^34^^,^^35^ This process should cover all aspects of decision making, including assessment of fitness, likelihood of success, and patient prioritisation.

### Maximising patient safety and learning curve

The Endo-Bentall remains very much in its infancy. Nonetheless, steps should be taken to ensure that operators are appropriately prepared. Use of cadaver and or animal models for simulation, visiting institutions where the Endo-Bentall has been performed successfully, and centralisation of this procedure can all be used to maximise the likelihood of success and mitigate the impact of the learning curve when performing a new procedure.^31^ Given the demonstrated safety and efficacy of proctor involvement in complex endovascular aortic procedures, proctor assistance may also be considered in developing Endo-Bentall programmes.^36^

### Oversight

Compassionate use typically refers to the use of an unproven device or procedure with the primary aim of producing a therapeutic benefit. This differs from research, where the goal is to produce generalisable data on the safety or efficacy of an intervention. However, this distinction is often not so dichotomous. Information and outcomes derived from compassionate use can be used to benefit future patients, in the same way that research frequently generates therapeutic benefit for participants. The two are therefore differentiated based on the clinician’s intent.^32^ Interestingly, although clinical research is subject to review and approval by Research Ethics Committees, compassionate care typically is not.^35^ The main argument supporting this practice is that the role of Research Ethics Committees is traditionally to protect the rights of patients participating in research, and not those undergoing treatment for therapeutic benefit.^37^ However, this creates potential loopholes that may be susceptible to exploitation. Indeed, some posit that data collection is specifically avoided in the setting of compassionate care to avoid the stringent approval process required for conducting research.^38^ This is problematic because it deprives future practitioners of valuable information that could be used to enhance decision making and improve safety. Furthermore, it may encourage selective reporting of positive outcomes while minimising or overlooking complications.^39^

**Figure 3:**
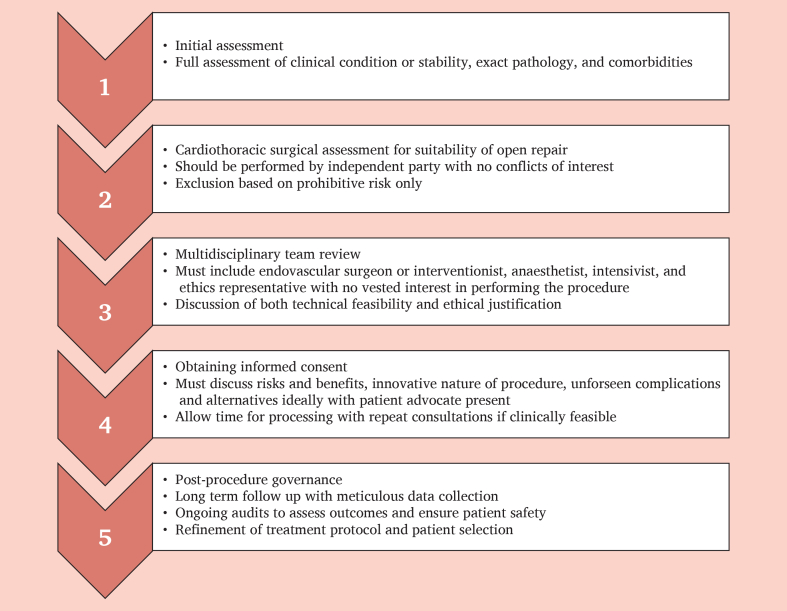
Five steps forming the process for initiation a novel surgical procedure such as Endo-Bentall in clinical practice.

Given these factors, the assessment by Borysowski *et al.*^37^ that compassionate use in the setting of the Endo-Bentall should be subject to standardisation, with consideration of ethical review regarding data collection and sharing of results of such compassionate procedures, is supported. The recommendations are summarised in Figure 3.

In terms of procedure development, there may be a point at which compassionate use transitions into clinical innovation. Although this threshold remains poorly defined, repeated application of the Endo-Bentall would increasingly move the procedure beyond the scope of individual compassionate use. At this stage, more robust governance mechanisms become necessary. Frameworks such as Idea, Development, Exploration, Assessment, Long term study (IDEAL) advocate structured evaluation through prospective registries, transparent reporting of technical modifications and complications, and ongoing refinement of patient selection criteria and procedure requirements.^40^

### Conclusion

Blindly labelling any patient declined for open repair as a compassionate case fit for the Endo-Bentall, or indeed any novel high risk procedure is ethically problematic. Significant harm can come to patients if surgical innovation is allowed to run rampant. An ethical analysis of different patients presenting with conditions amenable to treatment with the Endo-Bentall suggests that there is ethical justification for its use in selected patients depending on the clinical situation.

## Data availability

Data sharing is not applicable to this article as no datasets were generated or analysed.

## Use of Generative AI Tools

Generative artificial intelligence (AI) and artificial intelligence–assisted technologies were not used in the preparation of this work.

## Conflicts of interest

Anders Wanhainen: Institutional research grant from Cook Medical Inc and Gore Medical Inc. Rickard Lindblom: Speaker’s fee from Medtronic. Stefan James: Institutional research grant from Medtronic, Edwards, and Abbot and personal proctoring fees from Medtronic. Carlota Fernandez Prendes: Institutional research grant from Cook Medical Inc and Gore Medical Inc. Kevin Mani: Institutional research grant from Cook Medical Inc and Gore Medical Inc; consulting and speaker’s fee from Cook Medical Inc and Lombard Medical Inc.
